# Vitamin K Antagonists, Non–Vitamin K Antagonist Oral Anticoagulants, and Vascular Calcification in Patients with Atrial Fibrillation

**DOI:** 10.1055/s-0038-1675578

**Published:** 2018-11-10

**Authors:** Frederique E. C. M. Peeters, Elton A. M. P. Dudink, Dorien M. Kimenai, Bob Weijs, Sibel Altintas, Luuk I. B. Heckman, Casper Mihl, Leon J. Schurgers, Joachim E. Wildberger, Steven J. R. Meex, Bas L. J. H. Kietselaer, Harry J. G. M. Crijns

**Affiliations:** 1 Department of Cardiology, Maastricht University Medical Center+ and CARIM, Maastricht, The Netherlands; 2 Department of Clinical Chemistry, Maastricht University Medical Center+, Maastricht, The Netherlands; 3 Department of Radiology and Nuclear Medicine, Maastricht University Medical Center+ and CARIM, Maastricht, The Netherlands; 4 Department of Biochemistry, Maastricht University and CARIM, Maastricht, The Netherlands; 5 Department of Cardiology, Zuyderland Medical Center, Heerlen/Sittard, The Netherlands

**Keywords:** cardiac events, computed tomographic imaging, aortic and arterial diseases, atrial fibrillation, oral anticoagulant treatment

## Abstract

**Background** Vitamin K antagonists (VKAs) are associated with coronary artery calcification in low-risk populations, but their effect on calcification of large arteries remains uncertain. The effect of non–vitamin K antagonist oral anticoagulants (NOACs) on vascular calcification is unknown. We investigated the influence of use of VKA and NOAC on calcification of the aorta and aortic valve.

**Methods** In patients with atrial fibrillation without a history of major adverse cardiac or cerebrovascular events who underwent computed tomographic angiography, the presence of ascending aorta calcification (AsAC), descending aorta calcification (DAC), and aortic valve calcification (AVC) was determined. Confounders for VKA/NOAC treatment were identified and propensity score adjusted logistic regression explored the association between treatment and calcification (Agatston score > 0). AsAC, DAC, and AVC differences were assessed in propensity score–matched groups.

**Results** Of 236 patients (33% female, age: 58 ± 9 years), 71 (30%) used VKA (median duration: 122 weeks) and 79 (34%) used NOAC (median duration: 16 weeks). Propensity score–adjusted logistic regression revealed that use of VKA was significantly associated with AsAC (odds ratio [OR]: 2.31; 95% confidence interval [CI]: 1.16–4.59; *p* = 0.017) and DAC (OR: 2.38; 95% CI: 1.22–4.67; *p* = 0.012) and a trend in AVC (OR: 1.92; 95% CI: 0.98–3.80; *p* = 0.059) compared with non-anticoagulation. This association was absent in NOAC versus non-anticoagulant (AsAC OR: 0.51; 95% CI: 0.21–1.21; *p* = 0.127; DAC OR: 0.80; 95% CI: 0.36–1.76; *p* = 0.577; AVC OR: 0.62; 95% CI: 0.27–1.40; *p* = 0.248). A total of 178 patients were propensity score matched in three pairwise comparisons. Again, use of VKA was associated with DAC ( *p* = 0.043) and a trend toward more AsAC ( *p* = 0.059), while use of NOAC was not (AsAC *p* = 0.264; DAC *p* = 0.154; AVC *p* = 0.280).

**Conclusion** This cross-sectional study shows that use of VKA seems to contribute to vascular calcification. The calcification effect was not observed in NOAC users.

## Introduction

Vitamin K antagonists (VKAs) and non–vitamin K antagonist oral anticoagulants (NOACs) are widely prescribed drugs for prophylaxis and treatment of thromboembolic events. Although VKAs are proven to be effective in preventing thromboembolic events, their potential adverse effect in promoting soft-tissue calcification has not been taken into account in clinical management. VKAs exert their antithrombotic effect through interference with vitamin K metabolism, which is not limited to vitamin K–dependent coagulation factors, but also all vitamin K–dependent proteins, including those involved in calcification. 1 Clinical studies confirm the association between VKA and vascular calcification. 2 3 4 Since vascular calcification is known to be a marker of increased cardiovascular morbidity and mortality, 5 VKA-induced calcification has become nonnegligible—certainly in initially low- to intermediate-risk populations. This holds even more since current guidelines recommend oral anticoagulant (OAC) treatment even in low-risk atrial fibrillation (AF) patients with only one stroke risk factor. 6

NOACs are a modern alternative to VKA since trials proved NOACs to be noninferior to VKA treatment for several indications. 7 NOACs exert their anticoagulant effect by direct inhibition of either factor IIa or factor Xa in the coagulation cascade. Thus, NOACs are hypothesized to lack the detrimental effect on calcification. 1 8

Therefore, the purpose of this study was to investigate the contribution of VKA treatment and NOAC treatment to vascular calcification of the aortic valve and the aorta in patients with AF. 1 2

## Methods

### Study Population

In this cross-sectional observational study, 236 patients with AF without a history of major adverse cardiac and cerebrovascular events (cardiac arrest, acute coronary syndrome, revascularization, or stroke) were included. Patients were divided in three groups according to type of anticoagulation: no oral anticoagulation, VKA (acenocoumarol; scan date: 2007–2010) or NOAC (rivaroxaban [ *n* = 59], and dabigatran [ *n* = 11], or apixaban [ *n* = 9]; scan date: 2011–2016). Part of this population (non-anticoagulant group and VKA group) has been described previously. 9 In short, patients with paroxysmal AF undergoing a cardiac multislice computed tomography (MSCT) scan were included and the PROCAM risk score was determined. Patients with significant and untreated cardiovascular disease (CVD) including (untreated) hypertension, diabetes mellitus or hypercholesteremia, coronary artery disease, chronic kidney disease, or heart failure were not included. For the purpose of this study, a similar strategy was used to include CT scans of patients with NOAC treatment. Additionally, patients in whom OAC therapy was initiated with VKA and changed to NOAC over the years were excluded. All patients underwent MSCT either for workup before an electrophysiological ablation or for general checkup for coronary artery disease and were in sinus rhythm during examination. Clinical information was obtained from the electronic hospital charts and the absolute 10-year risk of an acute coronary event (fatal of nonfatal myocardial infarction or acute coronary death) was determined using the PROCAM risk score. 10 This study was approved by the local institutional review board.

### Data Acquisition and Analysis of Cardiac Multislice Computed Tomography

A non–contrast-enhanced coronary calcium scan was performed in all patients as described previously. 9 CT scans were performed using a Philips Brilliance 64-slice MSCT scanner (Philips Healthcare, Best, the Netherlands), second-generation dual-source CT scanner (Somatom Definition Flash 2*128-slice; Siemens Healthineers, Forchheim, Germany), or third-generation dual-source CT scanner (Somatom Definition Force 2*192; Siemens Healthineers). CT scans of Philips Brilliance MSCT scanner were analyzed with dedicated EBW Heartbeat CS software (Philips Healthcare). CT scans of the Siemens Somatom Definition Flash or Force were assessed using source images on a dedicated workstation (Syngo.via; Siemens Healthineers). For quantitative assessment of aortic valve and ascending and descending aorta calcification, the Agatston score was determined using a 3-mm CT slice thickness and a detection threshold of ≥130 HU involving ≥1 mm ^2^ area/lesion (3 pixels). 11 The presence of calcification of the ascending aorta calcification (AsAC), descending aorta calcification (DAC), and aortic valve calcification (AVC) was defined as Agatston score greater than 0. Calcium localized above the origin of the right coronary artery to the end of scan range, or up to the origin of the brachiocephalic artery, was considered to be in the ascending aorta. Calcium present distal from the origin of the left subclavian artery up to the diaphragm was considered to be localized in the descending aorta. The Agatston score of each segment was calculated separately by two independent observers, both blinded to medical data. In case of ambiguity, consensus was reached by discussion.

### Echocardiography

An independent observer performed the echocardiography while subjects were lying in the left lateral decubitus position. Standard two-dimensional transthoracic echocardiography was performed, including M-mode, and Doppler echocardiography (Sonos 5500 and IE33; Philips Medical Systems, Andover, Massachusetts, United States) according to the guidelines of the European Association of Echocardiography. 12

### Statistical Analyses

Statistical analyses were performed using SPSS version 22 (IBM Corp, Armonk, New York, United States). Normally distributed continuous variables are expressed as mean ± standard deviation (SD) and compared using independent samples *t* -testing. Non-normally distributed continuous variables are expressed as median (interquartile range [IQR]) and compared using the Mann–Whitney *U* -test. Categorical variables are expressed as absolute numbers and percentages and tested using the *χ* ^2^ test or Fisher's exact test.

Patients were stratified according to type of treatment (non-anticoagulant, VKA, or NOAC), the effect on the primary outcome, and the presence of calcification in the ascending and descending aorta, and the aortic valve between the groups was investigated using propensity scores ( Supplementary Fig. S1 ). Since patients were not randomly assigned to type of treatment, we adjusted for factors favoring non-anticoagulant, VKA, or NOAC prescription using propensity score to reduce the selection bias and see the genuine effect of therapy on the primary outcome. Groups were compared in a pairwise manner, 13 and for every pair, two analyses were performed as previously described. First, variables (mentioned in Table 1 ) associated with VKA or NOAC treatment were identified by univariable logistic regression (retention level set at 0.1). Subsequently, propensity scores of the variables showing a significant relation with prescription of VKA or NOAC were calculated for each patient and integrated in the logistic regression models as a covariate with the presence of calcification in the ascending aorta, descending aorta, and the aortic valve as outcome variables. Second, a propensity score–matched analysis was performed for every pair, in which patients were propensity score matched to patients in the treatment group they were to be compared with, using the Greedy matching strategy. 13 14 Propensity scores were matched to the closest propensity score of a patient in the other treatment arm with a maximum difference of 5%. Matching was repeated until all patients were matched, or until the propensity scores differed greater than 5% between arms. The χ ^2^ test was performed to assess the differences in the presence of AsAC, DAC, and AVC.

**Table 1:** Baseline characteristics of the total population

	Total population ( *n* = 236)	Non-anticoagulant ( *n* = 86)	VKA ( *n* = 71)	NOAC ( *n* = 79)
Demographics				
Age (y)	58.3 ± 9.2	55.8 ± 9.1	57.9 ± 9.0	61.5 ± 8.5
Male sex	159 (67.4)	53 (61.6)	57 (80.3)	49 (62.0)
BMI (kg/m ^2^ )	27.1 ± 3.6	26.6 ± 3.3	27.5 ± 3.2	27.2 ± 4.2
Smoking	27 (11.4)	11 (12.8)	8 (11.3)	8 (10.1)
Positive family history (AMI)	33 (14.0)	9 (10.5)	7 (9.9)	17 (21.5)
Systolic blood pressure (mm Hg)	128.2 ± 12.9	125.5 ± 9.9	124.5 ± 10.7	134.4 ± 15.1
Fasting blood glucose (mmol/L)	5.44 ± 0.52	5.43 ± 0.43	5.31 ± 0.54	5.58 ± 0.56
Cholesterol (mmol/L)	5.40 ± 0.96	5.44 ± 0.90	5.47 ± 1.14	5.28 ± 0.83
Triglycerides (mmol/L)	1.68 ± 0.92	1.56 ± 0.82	1.79 ± 1.13	1.70 ± 0.78
LDL (mmol/L)	3.41 ± 0.83	3.55 ± 0.73	3.52 ± 0.99	3.16 ± 0.74
HDL (mmol/L)	1.27 ± 0.35	1.20 ± 0.28	1.21 ± 0.41	1.41 ± 0.34
Creatinine (μmol/L)	86.7 ± 13.9	86.2 ± 14.2	88.9 ± 12.6	85.6 ± 14.5
PROCAM risk score (%)	7.0 [7.0]	5.7 [7.6]	7.0 [9.3]	7.0 [6.0]
CHA _2_ DS _2_ -VASc score	1.0 [1.0]	1.0 [1.0]	1.0 [1.0]	1.0 [1.0]
Anticoagulation duration (wk)	NA	NA	122 [158]	16 [36]
AF duration (mo)	25 [70]	26 [72]	42 [71]	8 [24]
Medication				
Rhythm control	146 (61.9)	51 (59.3)	51 (71.8)	44 (55.7)
Rate control	120 (50.8)	41 (47.7)	40 (56.3)	39 (49.4)
ACE inhibitors	32 (13.6)	9 (10.5)	11 (15.5)	12 (15.2)
Angiotensin receptor blockers	54 (22.9)	16 (18.6)	18 (25.4)	20 (25.3)
Diuretics	33 (14.0)	8 (9.3)	12 (16.9)	13 (16.5)
Statins	37 (15.7)	13 (15.1)	13 (18.3)	11 (13.9)
Echocardiography				
LA dimension (mm)	41.1 ± 5.2	40.0 ± 5.1	42.5 ± 5.0	41.1 ± 5.2
LA volume (mL)	76.8 ± 23.6	69.5 ± 21.9	80.3 ± 21.6	81.9 ± 25.7
RA volume (mL)	60.8 ± 24.8	57.0 ± 18.5	62.2 ± 22.7	64.0 ± 32.5
IVS (mm)	8.6 ± 0.8	8.5 ± 0.8	8.7 ± 0.8	8.7 ± 0.8
PW (mm)	8.5 ± 0.8	8.5 ± 0.7	8.6 ± 0.8	8.5 ± 0.8
LVEF (%)	60.2 ± 6.0	60.9 ± 6.1	60.5 ± 6.5	59.1 ± 5.1

Abbreviations: ACE, angiotensin-converting enzyme; AF, atrial fibrillation; AMI, acute myocardial infarction; BMI, body mass index; HDL, high-density lipoprotein; IVS, interventricular septum; LA, left atrium; LDL, low-density lipoprotein; LVEF, left ventricular ejection fraction; PW, posterior wall; RA, right atrium.

Notes: Continuous variables are expressed as mean ± SD or median [IQR] depending on their distribution. Categorical variables are reported as *n* (%).

**Fig. 1:**
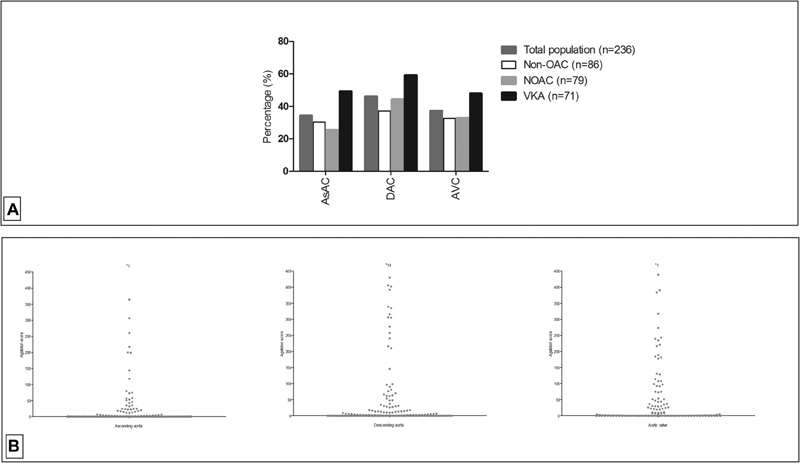
( **A** ) Presence of calcification in different segments in total population and according to treatment type. ( **B** ) Distribution of calcification (Agatston scores) in the ascending aorta, descending aorta, and aortic valve in the total population. (“*” indicates the number of values >500). AsAC, ascending aortic calcification; DAC, descending aortic calcification; AVC, aortic valve calcification; non-OAC, non-oral anticoagulant; VKA, vitamin K antagonist; NOAC, non–vitamin K antagonist oral anticoagulant.

The Jonckheere–Terpstra test was performed to assess whether duration of VKA treatment (in tertiles) was associated with the height of the total Agatston score in the VKA treatment group. A similar analysis could not be performed in NOAC users, due to the shorter duration of treatment in this group. Overall, statistical significance was assumed for *p* < 0.05.

## Results

### Baseline Variables

Mean age ( ±  SD) of the total population was 58 ± 9 years, 67% ( *n* = 159) were male and the median (IQR) CHA _2_ DS _2_ -VASc score was 1.0. Indications for MSCT were workup for ablation (51.5%) and general checkup for coronary artery disease. Further baseline characteristics are shown in Table 1 . AsAC, DAC, or AVC was present in 81 (34.3%), 109 (46.2%), and 88 (37.3%) patients, respectively ( Fig. 1A , B and Supplementary Fig. S2 ). A total of 47 (19.9%) patients showed calcification of both the aortic segments and the aortic valve. Of the total study population, 86 patients (36.4%) were in the non-anticoagulant group, 71 (30.1%) in the VKA group, and 79 (33.5%) in the NOAC group. Median durations of VKA and NOAC treatments were 122 (IQR: 51–209) and 16 (IQR: 6–42) weeks, respectively.

**Fig. 2:**
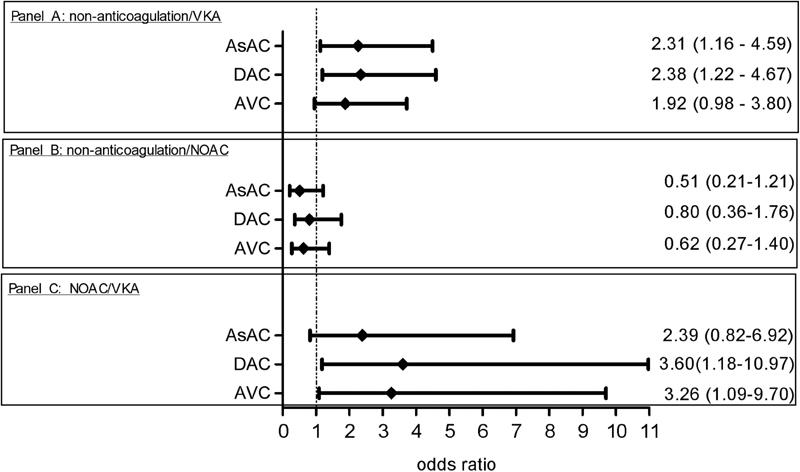
AsAC, DAC, and AVC in propensity score–adjusted regression analyses (odds ratio [95% CI]). ( **Panel A** ) Non-anticoagulation (reference) versus VKA. ( **Panel B** ) Non-anticoagulation (reference) versus NOAC. ( **Panel C** ) NOAC (reference) versus VKA. AsAC, ascending aortic calcification; DAC, descending aortic calcification; AVC, aortic valve calcification; VKA, vitamin K antagonist; NOAC, non–vitamin K antagonist oral anticoagulant.

### Identification of Confounders for VKA or Direct Oral Anticoagulant Treatment

Patients on VKA treatment were more likely to be male and treated with rhythm control medication than non-anticoagulant patients. Moreover, they were more likely to have a higher body mass index and larger left atrial diameters. Compared with the non-anticoagulant or VKA group, NOAC users were older, more likely to have higher systolic blood pressure, and their medical history with AF and thus anticoagulant treatment duration was shorter; however, they had a comparable left atrial volume to the VKA group. Additionally, they had a more favorable low-density lipoprotein and high-density lipoprotein cholesterol profile. In pairwise comparison between NOAC and VKA users, NOAC users were more likely to be of female sex and had slightly higher plasma glucose levels ( Supplementary Table S1 ).

### Calcification Differences between Types of Anticoagulation

Distribution of prevalence of calcification of all segments of the three groups is displayed in Fig. 1 .

Regression analyses, adjusted for the propensity scores to correct for the identified confounders, revealed that patients in the VKA treatment group had significantly more AsAC and DAC when compared with the non-anticoagulation group (AsAC: odds ratio [OR] 2.31, 95% confidence interval [CI] 1.16–4.59, *p* = 0.017; DAC: OR 2.38, 95% CI 1.22–4.67, *p* = 0.012) and showed a trend toward a higher calcium score of the aortic valve (AVC: OR 1.92, 95% CI 0.98–3.80, *p* = 0.059; Fig. 2A ). AsAC, DAC, and AVC were not significantly different between the non-anticoagulation and NOAC groups (AsAC: OR 0.51, 95% CI 0.21–1.21, *p* = 0.127; DAC: OR 0.80, 95% CI 0.36–1.76, *p* = 0.577; AVC: OR 0.62, 95% CI 0.27–1.40, *p* = 0.248, respectively; Fig. 2B ). Occurrence of DAC and AVC was significantly higher in the VKA group when compared with the NOAC group (DAC: OR 3.60, 95% CI 1.18–10.97, *p* = 0.025; AVC: OR 3.26, 95% CI 1.09–9.70, *p* = 0.034). AsAC was higher in the VKA treatment group as compared with the NOAC group, although a significant difference was not reached (AsAC: OR 2.39, 95% CI 0.82–6.92, *p* = 0.109; Fig. 2C ).

### Calcification Differences in the Propensity Score–Matched Population

A total of 178 patients were propensity score matched in three pairwise comparisons according to treatment type. For comparison between non-anticoagulation and the VKA group, 118 patients were matched (59 in each group). These numbers were 32 and 28 (16 and 14 per group) for the non-anticoagulation/NOAC and the NOAC/VKA comparisons, respectively. After the matching procedure, clinical conditions and echocardiographic measurements were identical ( Supplementary Table S2 ).

**Fig. 3:**
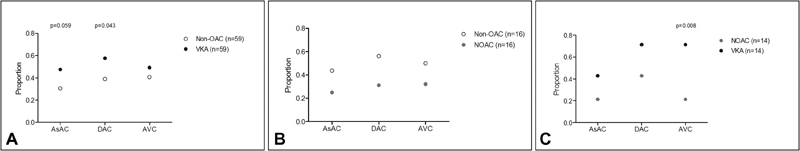
AsAC, DAC, and AVC per treatment group after propensity score matching. ( **A** ) Propensity score–matched cohort non-anticoagulation/VKA. ( **B** ) Propensity score–matched cohort non-anticoagulation/NOAC. ( **C** ) Propensity score–matched cohort NOAC/VKA. Statistical testing using chi-square. AsAC, ascending aortic calcification; DAC, descending aortic calcification; AVC, aortic valve calcification; non-OAC, non-anticoagulant; VKA, vitamin K antagonist; NOAC, non–vitamin K antagonist oral anticoagulant.

When comparing the presence of calcification between the propensity score–matched groups of non-anticoagulation and VKA, the presence of DAC remained significantly higher in the VKA-treated group ( *n* = 23 [39.0%] vs *n* = 34 [57.6%], *p* = 0.043), while AsAC showed a trend toward a higher presence ( *n* = 18 [30.5%] vs. *n* = 28 [47.5%], *p* = 0.059). AVC was not significantly different between the two groups ( *n* = 24 [40.7%] vs. *n* = 29 [49.2%] *p* = 0.355; Fig. 3A ).

The non-anticoagulation/NOAC-matched cohort did not reveal significant differences of AsAC, DAC, and AVC ( *n* = 7 [43.8%] vs. *n* = 4 [25.0%], *p* = 0.264; *n* = 9 [56.3%] vs. *n* = 5 [31.3], *p* = 0.154; *n* = 8 [50.0%] vs. *n* = 5 [31.3%], *p* = 0.280; Fig. 3B ). Less calcification in AsAC and DAC was seen in the NOAC group, although statistical significance was not reached ( *p* = 0.225 and *p* = 0.127); yet AVC was statistically significantly different between these groups ( *n* = 3 [21.4%] vs. *n* = 10 [71.4%], *p* = 0.008; Fig. 3C ).

### Vitamin K Antagonist Duration and Calcification

**Fig. 4:**
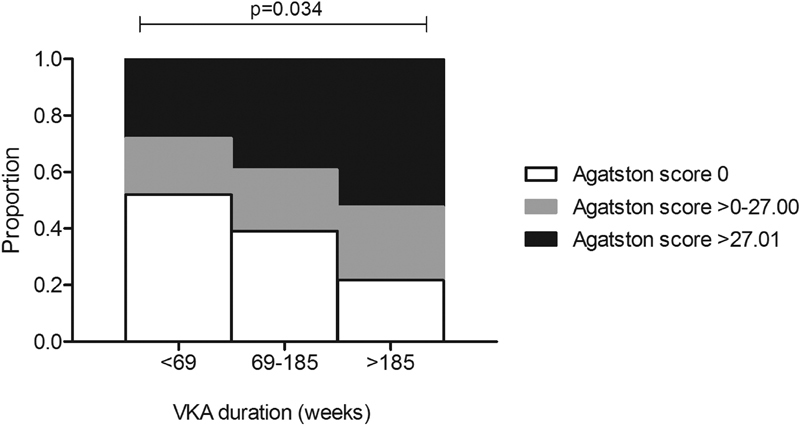
Vascular calcium score categories (tertiles) in patients with different treatment duration of vitamin K antagonist (VKA). Statistical testing using Jonckheere–Terpstra test.

Duration of treatment with VKA differed between patients. The Jonckheere–Terpstra test revealed a statistically significant trend between the extent of total vascular calcification and increased duration of VKA treatment ( *p* = 0.034). The rise in calcification levels and VKA duration is illustrated in Fig. 4 .

## Discussion

This study investigated the effect of anticoagulant treatment with VKAs and NOACs on vascular calcification as compared with patients with AF not treated with oral anticoagulation. The main finding of this study is that treatment with VKA is associated with a higher prevalence of calcification of the thoracic aorta when compared with patients without oral anticoagulation or NOAC treatment, whereas a difference in calcification between the NOAC and group without oral anticoagulation was not observed.

### Vitamin K Antagonists and Vascular Calcification

Several studies describing populations with variable CVD profiles reported an association between vascular and valvular calcification and VKA use. 2 3 4 Additionally, two studies considering relatively low-risk populations reported an association between femoral artery calcification and coronary artery calcification and VKA treatment. 9 15 Using propensity-matched analyses, we provide a funded direction to causality and show that in patients with similar risk profiles, those treated with VKA have more calcification of systemic arteries and the aortic valve in comparison to patients not treated with VKA. With that, our results are in accordance with previous data and extend knowledge, derived from these studies involving patients with different risk profiles, all treated with VKA. Although former studies showed controversial results, 2 16 we observed a trend in the duration of VKA treatment toward increased vascular calcification in the present population, hinting toward a direct calcification-inducing effect of VKA.

### The Mechanism Connecting Vitamin K Antagonist and Calcification

VKAs are established in the prevention of thromboembolic complications and exert their anticoagulant effect by inhibition of the activation of vitamin K–dependent coagulation factors II, VII, IX, and X. Bleeding is a major adverse effect of VKA and has been extensively described in literature. However, the calcification-induction potential of VKAs is not considered in its prescription in daily clinical practice. This effect on calcification is caused by the inhibition of matrix-γ-carboxyglutamic acid (Gla) protein (MGP), a vitamin K–dependent protein synthesized by vascular smooth muscle cells (VSMC). 17 The function of MGP as an inhibitor of soft-tissue calcification was shown in MGP-deficient mice which developed extensive arterial calcification and died within 2 months due to aortic rupture. 18 The effect of VKA on calcification was demonstrated in experimental animal models which showed similar development of calcification in vascular and aortic valve tissue as in MGP knockout animals. 19 The inhibition of MGP by VKA results in decreased calcium binding and inhibition of calcium crystals formation and bone morphogenetic protein action. 18 20 21 The loss of inhibitory function is furthermore caused by a relative shortage of vitamin K due to upregulation of MGP in areas with calcification, resulting in a high ratio of uncarboxylated versus carboxylated MGP. The last phenomenon is clinically associated with an increased risk for arterial and coronary calcification. 22 23 24

### VKA versus NOAC for Patients with Atrial Fibrillation

Aortic calcification is considered a subclinical marker of atherosclerosis, like coronary artery calcification, sharing risk factors with the latter. 25 The prognostic value of cardiovascular calcification has clearly been demonstrated in several patient populations with CVD, showing an increased risk of future events with increasing calcification. 26 27 The negative pharmacological characteristics and adverse events associated with the use of VKA opened the market for a new generation of anticoagulants (NOACs). 1 NOACs exert their anticoagulant effect by direct inhibition of factors IIa or Xa, and thus NOACs do not interfere with vitamin K–dependent proteins such as MGP. 17 In our study, we found no difference between the group of patients treated with NOACs as compared with those without oral anticoagulation. Furthermore, although treatment duration has to be taken into account, we found less calcification in the NOAC group in comparison to the VKA group. Thereby, although preliminary, we provide evidence that NOACs lack the calcification-induction effect of VKAs. The effect of both VKA and NOACs on both valvular and vascular calcification (including coronary artery calcification) is currently investigated in randomized controlled trials considering populations with different cardiovascular disease profiles. 28

Next to the suggested absence of this calcification inducing effect, direct beneficial effects of NOACs on attenuation of atherosclerosis and plaque stability have been suggested in animal models, 29 as NOACs inhibit PAR-receptor function through inhibition of factors IIa or Xa. Exemplary for this is that the COMPASS trial, investigating whether rivaroxaban alone or added to aspirin led to less cardiovascular events in comparison to aspirin alone, was halted after the interim analysis revealed superiority of the combined rivaroxaban plus aspirin in comparison to aspirin alone. 30 As AF is generally associated with an increased cardiovascular risk profile, this effect of NOACs can be expected to be of particular value in AF patients.

Overall, growing insight in the presence/absence of calcification-induction of different OACs and their effect on atherosclerosis holds potential to provide important implications to aid clinicians in their choice for anticoagulant treatment type, especially in patients with lifelong indication for OAC, such as patients with AF.

## Strengths and Limitations

Our study creates the opportunity to assess the true effect of the anticoagulation type in patients with a comparable cardiovascular profile by excluding several potential variables that could have influenced this effect, providing a funded direction toward a causal relationship. We believe that our results are of additive value due to the method of analyses using propensity score matching. Although the current study provides important insight, some caution must be considered with the interpretation. First, the sample size of the study resulted in relatively small propensity-matched arms of non-anticoagulation/NOAC and NOAC/VKA. Trials with a higher number of patients should be performed to overcome issues regarding the power in this study. Second, we tried to circumvent limitations generally involved in studies with a retrospective nature and a cross-sectional design. However, the design of the current study does not allow correcting for unmeasured confounders, whereby residual confounding could persist. Furthermore, levels of calcification before initiation of anticoagulation are unknown, and may have differed between patients. Also, the overall quality of anticoagulation control for patients treated with VKA could not be determined reliably. At last, the shorter duration of NOAC treatment in the current study has to be taken into account. Therefore, this result has a rather basic nature regarding the true (absent) effect of NOACs on calcification. Extrapolation of these results to a broader patient population should be done with caution. Studies on long-term use of NOAC are necessary to confirm these findings and to provide valuable additional information in the determination of a true “absent” effect of NOACs on calcification.

## Conclusion

Vascular calcification is known to be a marker of increased cardiovascular morbidity and mortality, making it nonnegligible, certainly in initially low-risk populations. All studies performed so far suggest vascular calcification, but could not define true causality between VKA and aortic and valvular calcification due to their design. Yet in this study, we show that VKA contributes to the presence of vascular calcification, which is not observed in NOAC. To address the question whether VKAs cause vascular calcification and NOACs do not, randomized controlled trials are on their way.
