# Supplementary material for: Vitamin K Antagonists, Non–Vitamin K Antagonist Oral Anticoagulants, and Vascular Calcification in Patients with Atrial Fibrillation
Source: TH Open. 2018 Nov 10;2(4):e391–8. doi: 10.1055/s-0038-1675578 (PMC6524908; doi:10.1055/s-0038-1675578)
Supplement: Supplementary file 1 — Supplementary Material [file 10-1055-s-0038-1675578-s180039.pdf]

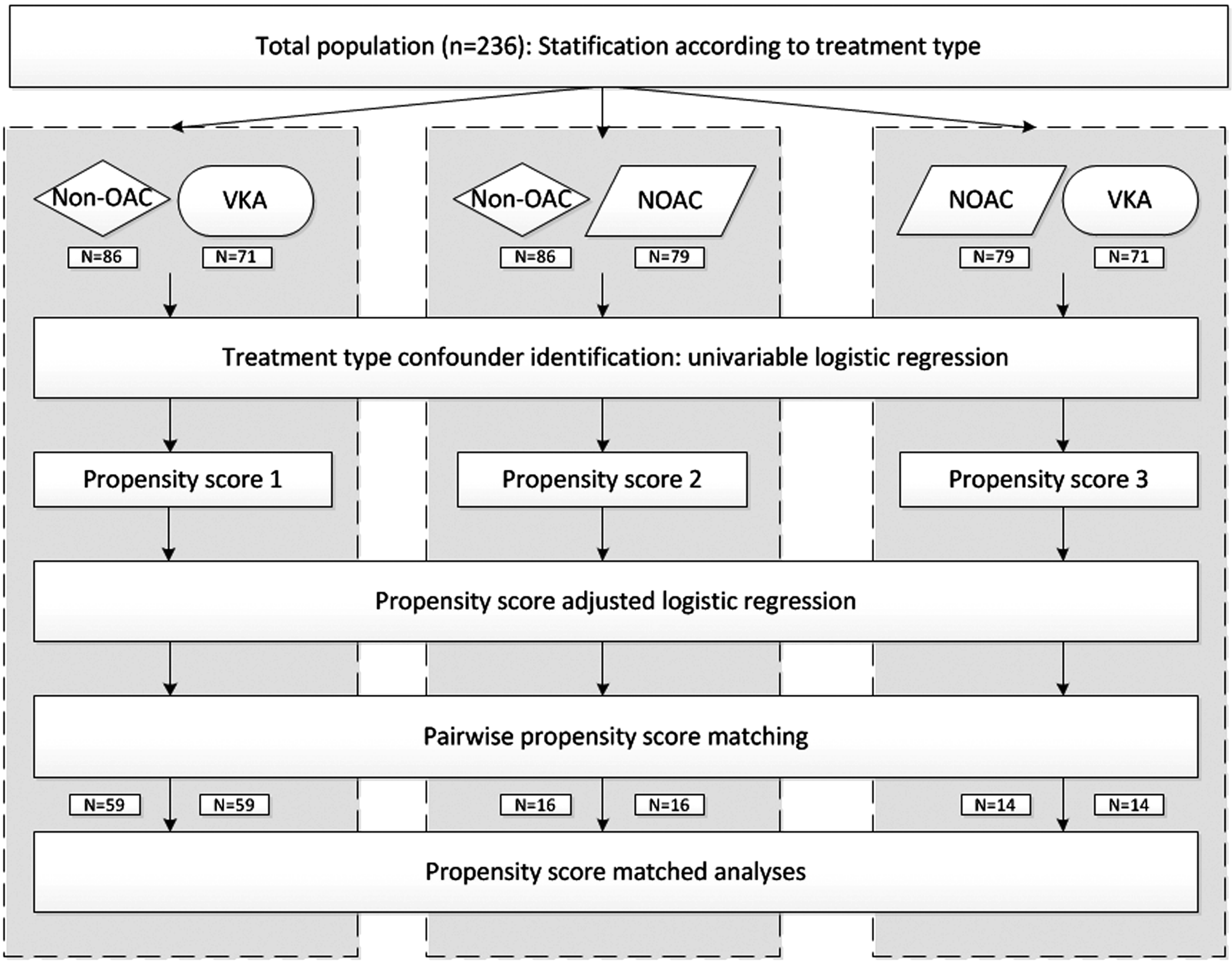

Supplementary Fig. S1 Flowchart of statistical analyses. Abbreviations: NOAC, non–vitamin K antagonist oral anticoagulant; non-OAC, non-oral anticoagulant; VKA, vitamin K antagonist.

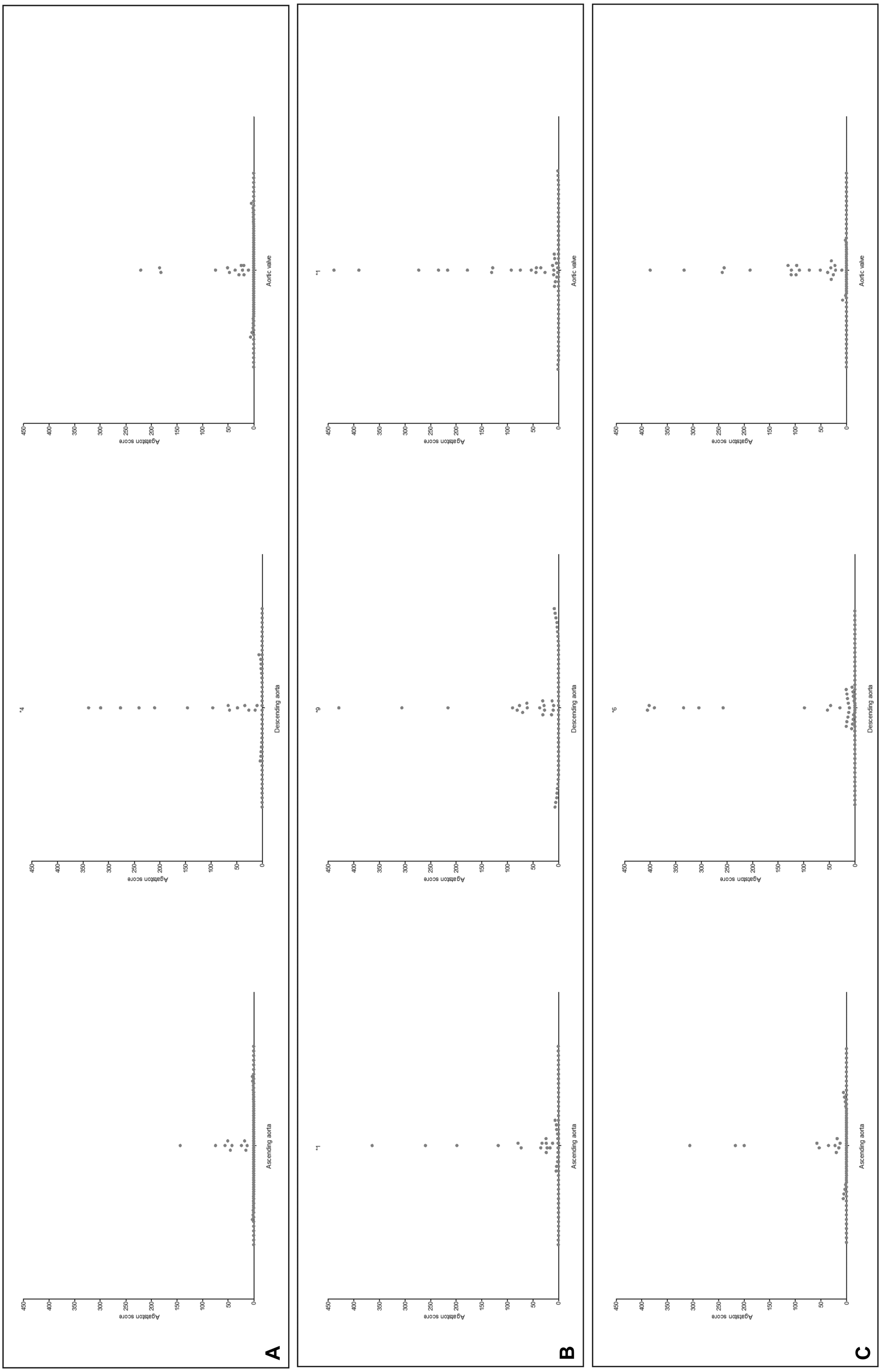

Supplementary Fig. S2 Distribution of calcification (Agatston scores) in the ascending aorta, descending aorta and aortic valve. (**A**) Non-OAC population. (**B**) VKA population. (**C**) NOAC population. ("*" indicates the number of values >450).

Supplementary Table S1 Characteristics of non-anticoagulant, VKA, and NOAC group

	Treatment groups			*p*-Value (95% CI for the difference)		
	Non-anticoagulant ($n = 86$)	VKA ($n = 71$)	NOAC ($n = 79$)	Non-anticoagulant vs. VKA	Non-anticoagulant vs. NOAC	NOAC vs. VKA
**Demographics**						
Age (y)	55.8 ± 9.1	57.9 ± 9.0	61.5 ± 8.5	0.157 (0.99–1.06)	<0.001 (1.037–1.120)	0.014 (0.917–0.990)
Male sex	53 (61.6)	57 (80.3)	49 (62.0)	0.012 (0.19–0.82)	0.958 (0.524–1.844)	0.016 (0.191–0.841)
BMI (kg/m^2)	26.6 ± 3.3	27.5 ± 3.2	27.2 ± 4.2	0.096 (0.99–1.20)	0.292 (0.963–1.135)	0.677 (0.934–1.111)
Smoking	11 (12.8)	8 (11.3)	8 (10.1)	0.849 (0.34–2.43)	0.943 (0.361–2.579)	0.912 (0.328–2.706)
Positive family history (AMI)	9 (10.5)	7 (9.9)	17 (21.5)	0.964 (0.36–2.97)	0.003 (1.569–9.657)	0.008 (0.100–0.712)
Systolic blood pressure (mm Hg)	125.5 ± 9.9	124.5 ± 10.7	134.4 ± 15.1	0.554 (0.96–1.02)	<0.001 (1.030–1.087)	<0.001 (0.918–0.970)
Fasting blood glucose (mmol/L)	5.43 ± 0.44	5.31 ± 0.54	5.58 ± 0.56	0.123 (0.30–1.15)	0.063 (0.968–3.420)	0.005 (0.207–0.754)
Cholesterol (mmol/L)	5.44 ± 0.90	5.47 ± 1.14	5.28 ± 0.83	0.878 (0.74–1.43)	0.280 (0.544–1.193)	0.291 (0.848–1.732)
Triglycerides (mmol/L)	1.56 ± 0.82	1.79 ± 1.13	1.70 ± 0.78	0.182 (0.89–1.85)	0.317 (0.811–1.908)	0.618 (0.758–1.593)
LDL (mmol/L)	3.55 ± 0.73	3.52 ± 0.99	3.16 ± 0.74	0.819 (0.66–1.39)	0.002 (0.302–0.756)	0.016 (1.093–2.386)
HDL (mmol/L)	1.20 ± 0.28	1.21 ± 0.41	1.41 ± 0.34	0.951 (0.41–2.58)	<0.001 (3.355–39.23)	0.002 (0.069–0.538)
Creatinine concentration (μmol/L)	86.2 ± 14.2	88.9 ± 12.6	85.6 ± 14.5	0.271 (0.99–1.04)	0.773 (0.974–1.019)	0.166 (0.993–1.044)
OAC duration (wk)	NA	122 [158]	16 [36]	NA	NA	<0.001 (1.013–1.030)
AF duration (mo)	26 [72]	42 [71]	8 [24]	0.123 (1.00–1.01)	0.006 (0.983–0.997)	<0.001 (1.008–1.025)
**Medication**						
Rhythm control	51 (59.3)	51 (71.8)	44 (55.7)	0.103 (0.89–3.43)	0.640 (0.465–1.601)	0.042 (1.026–4.010)
Rate control	41 (47.7)	40 (56.3)	39 (49.4)	0.280 (0.75–2.66)	0.828 (0.581–1.972)	0.394 (0.695–2.519)
ACE inhibitors	9 (10.5)	11 (15.5)	12 (15.2)	0.346 (0.61–4.05)	0.380 (0.600–3.813)	0.930 (0.428–2.534)
Angiotensin receptor blockers	16 (18.6)	18 (25.4)	20 (25.3)	0.304 (0.70–0.320)	0.317 (0.695–3.075)	0.956 (0.488–2.136)
Diuretics	8 (9.3)	12 (16.9)	13 (16.5)	0.158 (0.77–5.19)	0.182 (0.740–4.854)	0.911 (0.444–2.483)
Statins	13 (15.1)	13 (18.3)	11 (13.9)	0.587 (0.54–2.94)	0.804 (0.376–2.136)	0.443 (0.587–3.388)
**Echocardiography**						
LA dimension (mm)	40.0 ± 5.1	42.5 ± 5.0	41.1 ± 5.2	0.003 (1.04–1.18)	0.144 (0.985–1.111)	0.103 (0.989–1.125)
LA volume (mL)	69.5 ± 21.9	80.3 ± 21.6	81.9 ± 25.7	0.007 (1.01–1.04)	0.004 (1.007–1.039)	0.706 (0.982–1.012)
RA volume (mL)	57.0 ± 18.5	62.2 ± 22.7	64.0 ± 32.5	0.159 (1.00–1.03)	0.149 (0.996–1.027)	0.729 (0.984–1.011)
IVS (mm)	8.5 ± 0.8	8.7 ± 0.8	8.7 ± 0.8	0.234 (0.86–1.90)	0.339 (0.813–1.822)	0.838 (0.700–1.552)
PW (mm)	8.5 ± 0.7	8.6 ± 0.8	8.5 ± 0.8	0.236 (0.84–2.00)	0.593 (0.731–1.730)	0.559 (0.737–1.757)
LVEF (%)	60.9 ± 6.1	60.5 ± 6.5	59.1 ± 5.1	0.663 (0.94–1.04)	0.044 (0.891–0.998)	0.150 (0.985–1.105)

Abbreviations: 95% CI, 95% confidence interval; AF, atrial fibrillation; BMI, body mass index; HDL, high density lipoprotein; LA, left atrium; LDL, low density lipoprotein; LVEF, left ventricular ejection fraction; NA, not applicable; OAC, oral anticoagulation; PW, posterior wall; RA, right atrium.

Notes: Continuous variables are expressed as mean ± SD or median [IQR] depending on their distribution. Categorical variables are reported as *n* (%).

Supplementary Table S2 Propensity score–matched populations of non-anticoagulant/VKA, non-anticoagulant/NOAC, and VKA/NOAC

	Non-anticoagulant (n = 59)	VKA (n = 59)	p-Value (95% CI for the difference)	Non-anticoagulant (n = 16)	NOAC (n = 16)	p-Value (95% CI for the difference)	VKA (n = 14)	NOAC (n = 14)	p-Value (95% CI for the difference)
**Demographics**									
Age (y)	55.5 ± 9.4	57.6 ± 9.4	0.229 (−5.50; 1.33)	57.1 ± 7.6	54.8 ± 8.3	0.429 (−3.48 to 7.98)	59.7 ± 9.1	60.4 ± 6.1	0.386 (−9.04 to 3.61)
Male sex	45 (76.3)	45 (76.3)	>0.999	9 (56.3)	14 (87.5)	0.113	10 (71.4)	10 (71.4)	>0.999
BMI (kg/m^2)	27.4 ± 3.0	27.0 ± 3.1	0.420 (−0.65; 1.55)	26.3 ± 3.7	27.4 ± 4.4	0.438 (−4.06 to 1.80)	25.4 ± 3.3	28.6 ± 4.2	0.261 (−1.22 to 4.32)
Smoking	6 (10.2)	7 (11.9)	0.772	1 (6.3)	3 (18.8)	0.279	2 (14.3)	1 (7.1)	>0.999
Positive family history (AMI)	5 (8.5)	6 (10.2)	0.752	3 (18.8)	4 (25.0)	0.378	2 (14.3)	5 (50.0)	0.169
Systolic blood pressure (mm Hg)	124.2 ± 8.0	124.2 ± 10.6	0.992 (−3.40; 3.43)	130.0 ± 9.7	122.4 ± 14.3	0.088 (−1.20 to 16.45)	128.5 ± 10.1	135.9 ± 15.6	0.346 (−4.98 to 13.69)
Fasting blood glucose (mmol/L)	5.46 ± 0.42	5.33 ± 0.57	0.136 (−0.04; 0.32)	5.44 ± 0.47	5.75 ± 0.63	0.129 (−0.71 to 0.09)	5.40 ± 0.74	5.68 ± 0.67	0.352 (−0.25 to 0.69)
Cholesterol (mmol/L)	5.50 ± 0.92	5.49 ± 1.11	0.977 (−0.38; 0.40)	5.49 ± 1.00	5.44 ± 0.79	0.905 (−0.67 to 0.75)	5.29 ± 0.71	5.65 ± 0.82	0.204 (−0.22 to 0.97)
Triglycerides (mmol/L)	1.66 ± 0.91	1.79 ± 1.19	0.541 (−0.53; 0.28)	1.76 ± 1.12	2.07 ± 1.00	0.443 (−1.16 to 0.52)	1.73 ± 0.81	2.22 ± 0.90	0.797 (−0.65 to 0.84)
LDL (mmol/L)	3.55 ± 0.77	3.54 ± 0.95	0.959 (−0.31; 0.32)	3.49 ± 0.69	3.22 ± 0.51	0.219 (−0.017 to 0.71)	3.00 ± 0.80	3.37 ± 0.81	0.241 (−0.24 to 0.90)
HDL (mmol/L)	1.18 ± 0.30	1.20 ± 0.42	0.761 (−0.15; 0.11)	1.21 ± 0.29	1.30 ± 0.29	0.391 (−0.30 to 0.12)	1.50 ± 0.63	1.30 ± 0.25	0.870 (−0.34 to 0.40)
Creatinine concentration (µmol/L)	88.2 ± 12.6	87.5 ± 11.9	0.759 (−4.13; 5.64)	85.3 ± 17.0	91.6 ± 10.6	0.244 (−17.1 to 4.56)	93.1 ± 15.0	93.5 ± 15.2	0.504 (−8.43 to 16.70)
OAC duration (wk)	NA	119 [156]	NA	NA	20 [37]	NA	49.5 [72]	40.0 [53]	0.541
AF duration (mo)	36.0 [90]	41.0 [70]	0.372	49 [72]	8 [63]	0.468	25.0 [31]	20.8 [24]	0.874
**Medication**									
Rhythm control	42 (71.2)	40 (67.8)	0.842	10 (62.5)	8 (50.0)	0.476	9 (64.3)	9 (64.3)	>0.999
Rate control	27 (45.8)	34 (57.6)	0.269	9 (56.3)	6 (37.5)	0.288	6 (42.9)	7 (50.0)	0.705
ACE inhibitors	4 (6.8)	6 (10.2)	0.743	3 (18.8)	3 (18.8)	>0.999	2 (14.3)	3 (21.4)	>0.999
Angiotensin receptor blockers	12 (20.3)	17 (28.8)	0.391	3 (18.8)	3 (18.8)	>0.999	3 (21.4)	3 (21.4)	>0.999
Diuretics	5 (8.5)	11 (18.6)	0.177	1 (6.3)	2 (12.5)	>0.999	4 (28.6)	2 (14.3)	0.385
Statins	8 (13.6)	11 (18.6)	0.617	4 (25.0)	1 (6.3)	0.333	2 (14.3)	2 (14.3)	>0.999

Supplementary Table S2 (*Continued*)

	Non-anticoagulant ($n = 59$)	VKA ($n = 59$)	*p*-Value (95% CI for the difference)	Non-anticoagulant ($n = 16$)	NOAC ($n = 16$)	*p*-Value (95% CI for the difference)	VKA ($n = 14$)	NOAC ($n = 14$)	*p*-Value (95% CI for the difference)
Echocardiography									
LA dimension (mm)	41.5 ± 4.6	41.3 ± 4.4	0.774 (−1.40; 1.87)	38.4 ± 4.9	40.4 ± 6.2	0.335 (−5.97 to 2.10)	43.3 ± 6.9	41.8 ± 6.8	0.350 (−7.12 to 2.61)
LA volume (mL)	74.8 ± 22.5	78.0 ± 21.3	0.463 (−11.74; 5.39)	69.9 ± 19.7	78.2 ± 25.0	0.336 (−25.7 to 9.08)	82.9 ± 23.3	78.8 ± 20.4	0.574 (−23.59 to 13.42)
RA volume (mL)	61.5 ± 17.3	61.3 ± 23.3	0.128 (−8.18; 8.44)	55.9 ± 23.4	61.5 ± 26.4	0.556 (−24.9 to 13.7)	73.5 ± 24.0	62.9 ± 27.7	0.276 (−36.08 to 10.89)
IVS (mm)	8.6 ± 0.7	8.6 ± 0.9	>0.999 (−0.29; 0.29)	8.4 ± 0.9	8.7 ± 0.7	0.325 (−0.089 to 0.31)	8.7 ± 1.3	8.7 ± 0.7	0.730 (−0.90 to 0.64)
PW (mm)	8.6 ± 0.6	8.5 ± 0.7	0.286 (−0.12; 0.39)	8.4 ± 1.0	8.8 ± 0.6	0.146 (−1.01 to 0.16)	8.8 ± 0.9	8.5 ± 0.9	0.509 (−0.87 to 0.45)
LVEF (%)	61.0 ± 6.0	60.9 ± 6.2	0.952 (−2.15; 2.28)	61.8 ± 6.0	57.4 ± 6.2	0.051 (−0.03 to 8.78)	58.9 ± 6.6	57.4 ± 4.6	0.509 (−0.87 to 0.45)

Abbreviations: AF, atrial fibrillation; BMI, body mass index; HDL, high density lipoprotein; LA, left atrium; LDL, low density lipoprotein; LVEF, left ventricular ejection fraction; OAC, oral anticoagulation, PW, posterior wall; RA, right atrium.

Notes: Continuous variables are expressed as mean ± SD or median [IQR] depending on their distribution. Categorical variables are reported as *n* (%).
